# Land use and climate change-based multi-scenario simulation of ecosystem service trade-offs/synergies: A case study of the central Yunnan urban agglomeration, China

**DOI:** 10.1371/journal.pone.0324015

**Published:** 2025-06-25

**Authors:** Guoping Chen, Dandan Zhang, Junsan Zhao, Longjiang Zhang

**Affiliations:** 1 Chinese Academy of Surveying and mapping, Beijing, China; 2 Faculty of Land Resources Engineering, Kunming University of Science and Technology, Kunming, China; Saveetha Institute of Medical and Technical Sciences: Saveetha University, INDIA

## Abstract

Exploring Land use and climate change-based multi-scenario simulation of ecosystem service trade-offs/synergies is of great importance to regional ecological security and sustainable development. Taking the Central Yunnan Urban Agglomeration (CYUA) as a case study, six different scenarios of LULC-RCP were established to quantitatively assess four key ecosystem services(ESs) of water yield (WY), carbon stock (CS), soil conservation (SR) and habitat quality (HQ) with multiple objective programming and patch-generating land use simulation(MOP-PLUS) and integrated valuation of ecosystem services and tradeoffs (InVEST) models. The ESs were revealed regarding spatio-temporal trade-offs/synergies using Spearman correlation and geographically weighted regression (GWR). It was found that: (1)the ESs in CYUA is characterized with high spatial heterogeneity in 2030; specifically, the distribution of WY and SR was low in the northwestern region and high in the southeastern region, while the distribution of HQ and CS was high in the western region and the periphery, and low in the eastern and central regions; (2) the trade-offs between WY-HQ, and WY-CS, and the synergies between WY-SR, HQ-SR, HQ-CS, HQ-CS, and HQ-SR; (3) under the six different scenarios, the spatial distribution of trade-offs/synergies between the four ESs was consistent: the SR-HQ, SR-CS, and WY-CS showed an overall weak synergistic relationship; the HQ-CS showed an overall weak trade-offs; the HQ-WY, CS-WY showed an overall weak synergistic relationship in the northern and southern areas and an overall weak trade-off relationship in the center. The findings of this study may provide a theoretical foundation for ecosystem management in CYUA and offer technical support for the evaluation of national land space.

## Introduction

Ecosystem services (ESs) are the various direct or indirect products and services that humans obtain from the structure, functions, and processes of ecosystems, and is a bridge connecting human society and natural ecosystems [1]. Thus, the investigation on ESs is of great practical significance for improvement in human quality of life and regional sustainable development [2,3]. Due to the complexity of ecosystem services, spatial heterogeneity, and the diversity of human demands, the interactions between ecosystem services exhibit dynamic changes, forming trade-offs characterized by one increasing while the other decreases, or synergistic effects where both increase or decrease together [4,5]. The ESs changes are directly or indirectly affected by many factors of climate, soil, vegetation, and land type [3], among which, the changes in climatic environments and land-use patterns are two main factors driving changes in ESs and transitions between different types of ESs [6]. As a major driver of climate change, changes in atmospheric composition, e.g., increased concentrations of carbon dioxide, and other greenhouse gases such as nitrogen-containing compounds due to combustion and fertilization, can indirectly contribute to global warming by directly affecting biochemical processes and nutrient cycling in surface organisms [7]. Furthermore, sea level rise and changes in vegetation types due to global warming can in turn have a range of indirect effects on the bio-physical systems of the planet. Studies have demonstrated that climate change mainly featured global warming, has resulted in plenty of environmental issues like water scarcity, degradation of ESs, and increased risk of catastrophes [8], for example, about 60% of ESs have deteriorated because of climate change [9]. Since climate change has already severely affected the environment on which human beings depend and may further deteriorate in the future, it is becoming a significantly challenge for the sustainable development of human societies [10]. In addition, regional sustainable development is being seriously affected by deteriorating regional ESs due to intensification of human activities and ecological degradation. Overall, exploring the influences of land use (LC) and climate change on regional ESs and ESs trade-offs/ synergies to improve ecosystem management has become a global concern.

Land use/cover (LULC) change as a primary factor affecting ecosystem services [11], LULC change mainly affects the provision of ESs with changes in the internal structure and function of regional ecosystems by altering the physical properties of soils, vegetation cover, and LC [4]. Currently, the existing domestic and foreign researches on ESs trade-offs/synergies have been carried out with focus on the spatio-temporal differentiation, drivers, and scale effects [12,13]. For example, Mengba Liu et al argued that at different spatial scales, the impacts of LULC change and its coupling effects on ESs differed significantly among different levels of urban agglomerations(12); Chen et al explored the relationship between intensity of ESs and LULC change of counties in China from a spatial perspective, and the results showed significant spatial dependence and heterogeneity between the intensity of ESs and LULC change [14]. Climate change is a critical factors influencing ESs [15], which directly affects ecosystems with respect to the structure and function by altering the temperature and the frequency and intensity of precipitation, thereby affecting the capacity of providing ESs as well as the interactions and feedback mechanisms within ecosystems [16]. For example, in arid land biodiversity conservation services may be sacrificed in certain areas to maintain water availability [13]. It have been demonstrated the impacts of different climatic factors on ESs, among which the reduced precipitation and increased temperature are the main ones [17]. Consequently, it is vital to evaluate the influences of both climate change and land use on ESs and the ES trade-offs/synergies. Nevertheless, the main focuses of existing is the impacts of single factors (LULC or climate change) on ESs from a historical perspective, and is lack of attention on coupling effects of climate change and LULC, particularly such effects on ESs interactions under multiple scenarios in the future.

The influence of land use change (LUC) on ESs is manifested in the differences in ecosystem structure and function of ecosystems due to land use intensity and the direction of LUC. Currently, many researchers have investigated the influencing mechanisms of LUC on changes in ESs, using the tools like linear regression model, land transfer matrices, and scenario analyses [18–20]. Using a spatial linear regression model, Zhang et al investigated the spatial dependence of ecosystems on LUC in urban areas at different scales, providing practical guidance for urban development planning and environmental protection [21]. Using a transfer matrix in conjunction with a coupled coordination degree model, Sun et al investigated the impacts of LUC on ESs in terms of supply and demand in China and assessed the degree of coupling coordination between LUC and ESs at provincial scale [22]. In the course of these studies, many ES analysis models have emerged, such as the Ecosystem Service Value (ESV) [23,24], the Environmental Quality Index (EQI), and the Integrated Valuation of Ecosystem Services and Trade-offs (InVEST) model [25]. Among these models, the InVEST model enables quantitative analysis on spatial distribution and dynamic change of multiple ecosystem services, and thus has been widely employed for assessment on regional ESs [26]. However, most of the existing studies on the relationship between LUC and ESs were carried out with focus on single ES or overall ESs [27,28], and with less attention to multiple ESs. In addition, the dual pressure of global climate change and rapid urbanization has brought more uncertainty to ESs. Therefore, it is still an urgent issue to investigate the influencing mechanisms of LUC on ESs under multiple scenarios in the future, taking into account regional development needs.

In answer to global climate change, various global future scenario pathways have been devised by the Intergovernmental Panel on Climate Change (IPCC) to envisage a range of climate outcomes for human societies under different development approaches. For example, in the earlier Special report on emissions scenarios (SRES), Representative concentration pathways (RCPs), and Shared socioeconomic pathways (SSPs), a variety of trends that are likely to emerge during the development of societies and ecosystems in the 21st century have been depicted [29]. The establishment of the model provides researchers with criteria for prediction. In this paper, based on the latest IPCC Future Development Scenarios Framework (SSP-RCP), the future LC scenarios were combined with RCP scenarios to construct the MOP-PLUS-RCP coupled model to simulate future changes in ESs under various scenarios considering both climate and land use, which can help to estimate the uncertainty of changes in ESs and the ESs trade-offs/synergies, thereby facilitating the identification of hotspots by policymakers regarding the changes in ESs to formulate more effective environmental responses, and ultimately realizing green and healthy sustainable development.

Plateau urban agglomerations, which are usually featured with unique geography, climate, and ecology, play an vital role in China’s strategic layout and can act important nodes connecting domestic and foreign markets with their special geographical location and resource endowment, facilitating the socio-economic development of the regions along the plateau regions; and therefore they are an important fulcrum for implementing many national strategies in China like the Western Development, the Belt and Road Initiative and so on. At the same time, they are conducive to optimization of the urbanization structure and promotion of balanced regional development. However, they are facing many challenges for their special natural environment, climate change, and intensified human activities, including intricate water-sand dynamics, fragile ecosystems, and imbalance between human and environmental systems. Therefore, predicting changes in ESs and ESs trade-offs/synergies in the future for plateau urban agglomerations based on LC and climate change-based multi-scenario simulations is of great significance for formulating overall regulation policies for sustainable development to achieve sustainable development in plateau regions with harmony between people and the land. Given this, this study uses the Central Yunnan Urban Agglomeration (CYUA) as a case study and employs the constructed MOP-PLUS-RCP coupled model to simulate land use changes (LUC) under three scenarios for 2030: Natural Increase (NIS), Ecological Protection (EPS), and Economic Development (EDS). Additionally, six different LULC-RCP scenarios were set up in conjunction with two climate scenarios (RCP4.5 and RCP8.5). Secondly, the InVEST model was used to quantitatively assess future changes in four key ecosystem services (ESs): water yield (WY), carbon storage (CS), soil retention (SR), and habitat quality (HQ). Thirdly, Spearman correlation analysis and geographically weighted regression (GWR) methods were employed to deeply analyze the spatio-temporal trade-offs and synergies between various ecosystem services. Finally, the main development pathways and management policies for future ecosystem management were proposed.

The study aimed (1) to simulate the changes in and spatial distribution of land use demand in 2030 under various scenarios by coupling the MOP-PLUS model, (2) to evaluate the spatio-temporal changes of four key ESs under six LULC-RCP scenarios with the InVEST model in combination with future LC and climate scenarios, (3) to investigate the trade-offs/synergies between certain ESs under various LULC-RCP scenarios using Spearman correlation and GWR, (4) to propose main development pathways and management policies for ecosystem management in the future in CYUA, according to the changes in ESs and ESs trade-offs/synergies under certain scenarios. The study is to creatively consider both climate change and LC, with focus on the changes in multiple ESs and the trade-offs/synergies between them, to offer policy recommendations for determining preferential development pathways and improving the overall ecosystem quality. The results of the study offer new insights, theoretical basis, and strategic directions, which may contribute to the solution to climate change and the achievement of sustainable development in CYUA.

## Study area and data source

### Study area

**Fig 1:**
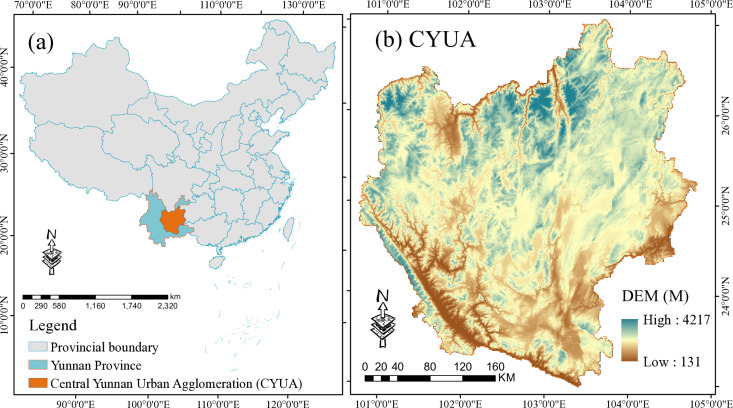
Research location map.

In the early 20th century, geographers and urban scholars began to focus on the interactions between cities, introducing concepts such as urban clusters to explain the spatial distribution patterns of cities and their interactions. In China, with the deepening of urbanization and regional economic development, urban agglomerations have become an important research field and practical issue for promoting national and local economic growth. CYUA is the city cluster in the most developed area of Yunnan Province (Fig 1), with a total area of 111,400 km^2^. It is one of the 19 city clusters in China with that the state focuses on cultivating, and the core area of productivity layout and production development in Yunnan Province. CYUA is dominated by mountains and basins, and belongs to the high altitude areas at low latitude. The region is rich in biological resources, with a forest cover of more than 50%, vegetation primarily dominated by subtropical evergreen broadleaf forests and scrublands & grasslands, and well-developed river systems, making it an important regional ecological barrier. Recently, as a result of accelerating urbanization, the demand on natural resources is growing, intensifying the conflict between environmental protection and urbanization as well as human activities, and the sustainable development in the region are facing multiple pressures and severe tests in terms of regional land and the ecological environment.

### Data sources and data processing

This study utilizes land use maps, digital elevation models, soil data, climate data, and socio-economic data. Table 1 summarizes the resolution and sources of each data type. Data preprocessing was carried out using ArcGIS software. Based on the 25 land use categories available in the data, we reclassified the land use data into cropland, forest, grassland, water bodies, built-up areas, and unused land. All data underwent preprocessing steps such as projection transformation, clipping, mask extraction, and resampling. ArcGIS was used to unify the coordinate system of all raster data, and the spatial resolution was standardized to 30 meters.

**Table 1:** Data sources.

Date name	Spatial Resolution	Time resolution	Date sources
**Land use Date**	30 m	2000、2010、2020	Resource and Environment Science and Data Center of the Chinese Academy of Sciences (http://www.resdc.cn/)
**DEM**	30 m	/	Geospatial data cloud (https://www.gscloud.cn/)
**Slope**	30m	/	Obtained from DEM calculations
**Soil type**	1km	/	Harmonized World Soil Database (https://www.fao.org/)
**Temperature**	1 km	2000、2010、2020	China Science and Technology Resources Sharing Service Platform-National Earth System Science Data Center (http://www.geodata.cn)
		2030	
**Precipitation**	1 km	2000、2010、2020	
		2030	
**Evapotranspiration**	1 km	2000、2010、2020	
		2030	
**Population density**	1 km	2000、2010、2020	Resource and Environment Science and Data Center of the Chinese Academy of Sciences (http://www.resdc.cn/)
**GDP**	1 km	2000、2010、2020	
«Yunnan Provincial Statistical Yearbook» (2000–2020)			Yunnan Provincial Government Website (http://stats.yn.gov.cn/)
«Yunnan Province Ecological Civilization Construction Leading Plan (2021–2025) »			
«Central Yunnan Urban Agglomeration Development Plan»			
«Yunnan Province Land Space Ecological Restoration Plan» (2021–2035)			

## Research methodology

### Projections of future climate and land use change scenarios

**Fig 2:**
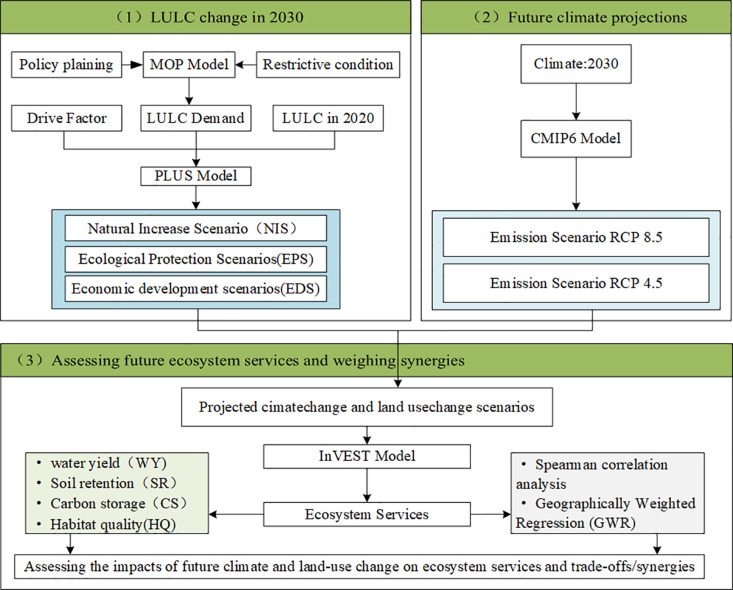
Study technical route.

This study takes CYUA as a case study and constructs the MOP-PLUS-RCP coupled model to predict different climate and land use scenarios for 2030. The Integrated Valuation of ESs and InVEST model was used to quantitatively assess the future changes in four key ESs: water yield (WY), carbon storage (CS), soil retention (SR), and habitat quality (HQ). Spearman’s correlation analysis and geographically weighted regression (GWR) were applied to explore the spatiotemporal trade-offs and synergistic effects among different ESs. Finally, the study proposes key development pathways and management strategies for future ecosystem management. The overall research framework is shown in Fig 2.

#### Climate data and emission scenarios.

To predict the distribution of future climate forcings, this study uses two Shared Socioeconomic Pathways (SSPs) from the latest CMIP6 updates: RCP 4.5 and RCP 8.5. RCPs are quantitative descriptions of alternative environmental development pathways for the 21st century, combining climate change projections with assumptions about mitigation or adaptation policies to generate comprehensive climate change scenarios. The RCP 4.5 scenario (4.5 W/m²) represents a moderate path for greenhouse gas emissions. In this scenario, environmental systems face some degree of degradation, and climate protection measures are assumed to be implemented [29]. The RCP 8.5 scenario (8.5 W/m²), on the other hand, represents the extreme case of rapid socioeconomic development, leading to the highest path for future greenhouse gas emissions [30]. In this scenario, global markets become increasingly integrated, fostering innovation and technological progress. However, socioeconomic development heavily relies on intensified exploitation of fossil fuel resources, with a higher proportion of coal, and an energy-intensive lifestyle continues globally [31]. This study selects RCP 4.5 and RCP 8.5 because they represent the moderate and worst-case scenarios for greenhouse gas emissions, respectively.

#### Land use change scenarios.

MOP is an open and flexible method, which mainly consists of three parts: decision variables, objective function, and constraints, and takes into account the manager’s expectations of relevant planning by setting proper objective optimization functions and constraints [32]. To investigate the trade-offs/synergies between ESs on the basis of simulated LUC under different scenarios, three LC scenarios of NIS, EPS and EDS were constructed herein in the light of the characteristics of LUC and compliance to the requirements of macro-policy regulation.

1. NIS: in this this scenario, the demand on LC by 2030 followed the same trend as the historical data, is not affected by policies, and serves as a reference baseline for other scenarios. The amount of various types of LC in 2030 under this scenario was calculated using the LUC matrix (2010–2020), which was generated by Markov model, and with various types of LC in 2020 as the baseline.
2. EPS: this scenario indicates that priority is given to the protection of ecological land (woodlands, grasslands, waters). In this scenario, the ecological benefits provided by each type of LC are maximized through planning and management, and the function for assessing ecological benefits is expressed as follows.

$$f_{1}(x)=\sum_{i=1}^{6}{ESV}_{i}\times x_{i}=0.5x_{1}+2.72x_{2}+1.55x_{3}+16.12x_{4}-1.58x_{5}+0.08x_{6}$$ (1)

In equation (1): f(x) denotes the total ecological benefit of all types of LC, ESV_i_ denotes the ecological benefit per hectare of the type i land use (in million CNY), and x_i_ denotes the area of the type i LC. In this paper, the economic value was obtained by correcting the economic value generated by the yield per unit area of the main crops in CYUA, with reference to the revised Equivalent Value per Unit Area of ESs in China by Xie et.al as the basis [33]. The objective optimization function of the EPS is: $max\{f_{1}(x\}$.

1. EDS: this scenario represents a great effort on economic development effort, so as to maximize the economic benefits to humans from various types of land, and the function for assessing economic benefits is given in equation (2) below.

$$f_{2}(x)=\sum_{i=1}^{6}{EC}_{i}\times x_{i}=12.88x_{1}+0.79x_{2}+7.74x_{3}+7.10x_{4}+785.85x_{5}+0.001x_{6}$$ (2)

In equation (2), f(x) denotes the total economic benefit, EC_i_ denotes the economic benefit per hectare of type i LC (in million CNY), and x_i_ denotes the area of type i LC. The average land economic value is sourced from the Yunnan Statistical Yearbook, where the total value of output for forestry, animal husbandry, and fishery corresponds to the economic benefits achieved by woodland, cropland, grassland, and waters, respectively. The GDP of the secondary and tertiary sectors corresponds to the economic benefits of built-up land, and the unused land was set based on the previous study (Ling et al., 2024). The objective optimization function of the EDS scenario is: $max\;\{f_{2}(x\}$.

The constraints are as follows.

1) Total land area constraint: It is necessary to ensure that the total area of all land use types remains unchanged. As shown in equation (3) below.

$$S_{total}=\sum_{i=1}^{6}=11141039.34\;(ha)$$ (3)

2) Total population constraint(*P*): this constraint ensures that the total population living on the land should not exceed the land’s carrying capacity for human activities. The upper limit of population was set according to the policy laid down in the 《Central Yunnan Urban Agglomeration Development Plan》(https://www.yn.gov.cn/), and the existing population is the lower limit. Therefore, the mathematical expression for the constraint on the total population can be written as equation (4).

2127≤P≤3050 (4)

In the formula, *P* represents the total population of the study area, measured in ten thousand people.

3) Cropland area constraint: the food yield of farmland should be at least the food demand of the population. Herein, the per capita food demand was assumed as 141.2 kg; the food self-sufficiency rate is 114.11 percent; the food production per hectare of the cropland was 4316.24 kg; the proportion of crops planted was 67 percent; and the multiple cropping index is 1.11. The cropland constraint can be written as follows:

$$P\times 141.2\times 114.11\;\%\leq x_{1}\times 4316.24\times 1.11\times 0.67$$ (5)

4) Forest area constraint: The area of forest land in the CYUC showed a downtrend from 2000 to 2020, which was expected to continue in the future. According to the ‘Yunnan Province Ecological Civilisation Construction Leading Plan (2021-2025)’ and the ‘Development Plan for the Central Yunnan Urban Agglomeration (CYUC)’, the local government scheduled to implement more ecological protection plans. Herein, using the woodland area in 2020 as the baseline, the land area (2020) ±1% was determined as the upper and lower bounds, respectively; and then the constraint can be written as:

$$5522431.16\leq x_{2}\leq 5413076.08$$ (6)

5) Grassland area constraint: a downtrend of grassland area was identified during the study, so using the grassland area projected by the NIS scenario as a baseline, the projected area ±1% was determined as the upper and lower bounds, respectively; and then the constraint can be written as:

$$2920662.98\leq x_{3}\leq 2879666.28$$ (7)

6) Water area constraint: Large areas of water in CYUA have been reclaimed for urbanization and crop production. To prevent further shrinkage of lake area, the local government has carried out the Dianzhong Diversion Project for water resources protection. As a result, the water area will be on an upward trend in the future. Therefore, the water area in 2020 was set as the lower bound of constraint, and the water area in 2030, which was calculated according to the natural growth rate of the water area from 2015 to 2020, was considered as the upper bound of the constraint, then the constraint can be written as:

$$146419.93\leq x_{4}\leq 164919.00$$ (8)

7) Built-up land constraint: the built-up land should satisfy the population demand and the per capita building land in CYUA was 0.0109 ha. Therefore, the constraint can be written as:

$$P\times 0.0109\leq x_{5}$$ (9)

However, the built-up area was projected to be 332,112.951 hectares after 2020 and 367,072.209 in 2030, based on the current trend of growth in built-up land in CYUA. Then the constraint can be detailed as:


$$332112.95\leq x_{5}\leq 36707.21$$ (10)

8) Unused land area constraint: since CYUA has been reclaiming unused land for long time due to socio-economic development, it is expected a smaller area of unused land in 2030 than that of 16,049.16 ha in 2020. However, since CYUA is a plateau zone, the utilization rate of unused land is low. Therefore, the unutilized land will not be less than 15849.3 ha. Then, the constraint can be written as:

$$15849.30\leq x_{6}\leq 16049.16$$ (11)

9) Other constraints: in view of the ecological protection measures proposed in the ‘Yunnan Province Ecological Civilisation Construction Leading Plan (2021-2025)’, the study concluded that the area of ecological land (woodland land, grassland, and water) in CYUA will be at least 75% of the total area by 2030. Therefore, the constraints can also be written as:

$$x_{2}+x_{3}+x_{4}\leq 0.75\times S_{total}$$ (12)

#### PLUS model.

As a raster data-based meta cellular automata (CA) model, the PLUS model can be used for simulation of patch-scale change in LULC [34]. The model integrates a land expansion analysis strategy-based rule mining framework (LEAS) and a multi-type random patch seeding-based CA model (CARS). Using the random forest classification (RFC) method, the proper probability for various types of LULC expansion can be obtained by mining the driving factors. It consists of two modules: (1) a rule mining framework based on Land Expansion Analysis Strategy (LEAS); (2) a Cellular Automata (CA) model based on multi-type random patch seeding (CARS). The steps are mainly as follows.

1. By using LEAS, the change patches of different land use types over a period can be analyzed to obtain the transition rules for all land use types. The transition rules for each land use type are mined and transformed into a binary classification problem, which allows for the calculation of the change probability and inertia probability for each land use type. Additionally, LEAS can employ the Random Forest Classification (RFC) algorithm to explore the relationship between the growth of different land use types and multiple driving factors.
2. The CARS module is a Cellular Automata (CA) model based on multi-type random patch seeding. The CA model is a scenario-driven land use simulation tool that integrates both “top-down” (i.e., overall land use demand) and “bottom-up” (i.e., regional land use competition) effects. During the simulation, land use demand influences land use competition through adaptive coefficients, thereby driving the land use amount to meet future demand (obtained through the MOP model in this study).

#### LULC-RCP multi-scenario model.

According to the needs of the study, six modelling scenarios, as shown in Table 2, were established by coupling the future climate models (RCP4.5, RCP8.5) and the three land use scenario data (NIS, EPS, EDS) in 2030. Among them: in Scenario 1, the data on LC under NIS scenario and the climate of RCP4.5 scenario in 2030were used as input data; in Scenario 2, the data on LC under NIS scenario and the climate of RCP8.5 scenario in 2030 were used as input data; in Scenario 3, the data on LC under EPS scenario and the climate of RCP4.5 scenario in 2030 were used as input data; in Scenario 4, the data on LC under EPS scenario and the climate of RCP8.5 scenario in 2030 were used as input data; in Scenario 5, the data on LC under EDS scenario and the climate of RCP4.5 scenario in 2030 were used as input data; and in Scenario 6, the data on LC under EDS scenario and the climate of RCP8.5 scenario in 2030 were used as input data.

**Table 2:** Future LULC-RCP multi-scenario models.

Situational model		Scenario 1(S1)	Scenario 2 (S2)	Scenario 3 (S3)	Scenario 4 (S4)	Scenario 5 (S5)	Scenario 6 (S6)
**Input data**	LULC	NIS	NIS	EPS	EPS	EDS	EDS
	Climate change	RCP4.5	RCP8.5	RCP4.5	RCP8.5	RCP4.5	RCP8.5

### Quantifying ESs

#### 1. WY.

The WY of the waters characterizes the regional water supply capacity, and during the experiment, in the InVEST model, the WY module was used for WY evaluation. The WY module is on the basis of the law of water balance, taking into account the factors of vegetation cover, climate, and soil texture, etc., and the primary formula is as follows:


$$Y_{x}=\left(1-\frac{{AET}_{x}}{P_{x}}\right)\times P_{x}$$ (13)

Where: $\backslash (Y_{x}\;\backslash )$denotes the W of each raster x (mm); $\backslash ({AET}_{x}\backslash )$ and $\backslash (P_{x}\backslash )$ denote the actual annual evapotranspiration and annual precipitation of each raster x (mm), respectively [17].

#### 2. CS.

In the InVEST model, the CS module divides the CS of EC into four parts: above-ground CS, below-ground CS, soil CS and dead organic matter CS. Using the module, the CS in the watershed was calculated herein with the following equation:


$$C_{total}=C_{above}+C_{below}+C_{soil}+C_{dead}$$ (14)

Where: $\backslash (C_{total}\backslash )$ denotes the total CS (t/hm^2^); $\backslash (C_{above}\backslash )$ denotes the above-ground CS (t/hm^2^); $\backslash (C_{below}\;\backslash )$denotes the below-ground CS (t/hm^2^); $\backslash (C_{soil}\backslash )$ denotes the soil CS (t/hm^2^); and C_dead__ denotes the dead organic matter CS (t/hm^2^). Herein, the data on carbon density for the various types of LC was sourced from the National Ecosystem Science Data Centre and certain literature [28,35,36]. The carbon density data were sourced from studies on various regions in China, thus the selected literatures should have a study area as close to or similar to that in this study as possible to prevent significant error due to data gap. In addition, carbon density varies depending on many factors like climate, soil properties and LC. Therefore, appropriate corrections should be made in accordance with the types of LC and the climate characteristics in different periods in CYUA. It has been reported a positive correlation of carbon density with annual precipitation and a weak correlation between carbon density and average annual temperature [37]. The specific calculation process refers to equations (3)–(9) in the literature [38], and the carbon densities in each period are shown in Annex 1.

#### 3. HQ.

In the InVEST model, the HQ module has been employed for calculating the HQ of upper basin of the Yangtze River, using the following formula. In this paper, cropland and built-up land were used as habitat stress factors, and the values of the required parameters were assigned with reference to the existing literature [16].


$$Q_{xj}=H_{j}\left(1-\left(\frac{D_{xj}^{z}}{D_{xj}^{z}-K^{z}}\right)\right)$$ (15)

$$\begin{array}{c} D_{xj}=\sum_{r=1}^{R}\sum_{y=1}^{Y_{r}}\left(\frac{W_{r}}{\sum_{r=1}^{R}W_{r}}\right)r_{y}i_{rxy}\beta_{x}S_{jr} \end{array}$$ (16)

where $\backslash (Q_{xj}\backslash )$ denotes the HQ of raster x; $\backslash (D_{xj}\backslash )$ denotes the total threat level of raster x; K and Z denote scaling factors; $\backslash (H_{j}\backslash )$ denotes the HQ of LULC type j; R denotes the coercion factor; $\backslash (Y_{r}\backslash )$ denotes the number of rasters occupied by the stress factor r; $\backslash (W_{r}\backslash )$ denotes the weight of the stress factor, within a range of 0–1; $\backslash (r_{y}\backslash )$ denotes the value of the coercion factor for raster y; $\backslash (i_{rxy}\backslash )$ denotes the coercion ($\backslash (r_{y}\backslash )$) of the raster y to the level of stress for raster x; $\backslash (\beta_{x}\backslash )$ denotes the level of accessibility for raster x; and $\backslash (S_{jr}\backslash )$ denotes the sensitivity of type j habitat to the stress factor r.

#### 4. SC.

Herein, the erosion model of Revised Universal Soil Loss Equation was used to estimate soil conservation in CYUC, using the following formula [39]:


$$\begin{array}{c} A=R\times K\times LS\times (1-C\times P) \end{array}$$ (17)

Where: A denotes the soil conservation per unit area; R denotes the factor of rainfall erosivity; K denotes the factor of soil erodibility; LS denotes the factor of slope length; C denotes the factor of vegetation cover and management; P denotes the factor of soil and water conservation measures.

### ESs trade-offs/ synergies

#### Spearman correlation analysis.

To detect the correlation between the ESs on the time scale, Spearman’s correlation analysis and significance test were performed based on the R platform using the ‘Hmisc’ package [40]. Positive and negative correlations indicate synergies and trade-offs between ESs, respectively; and the larger the absolute value of the correlation coefficient, the greater the relationship. The calculation formula is as follows [41]:


$$\begin{array}{c} \rho =\frac{\sum_{i=1}^{n}\left(X_{i}-\overset{―}{X}\right)\left(Y_{i}-\overset{―}{Y}\right)}{\sqrt{\sum_{i=1}^{n}{\left(X_{i}-\overset{―}{X}\right)}^{2}}\sqrt{\sum_{i=1}^{n}{\left(Y_{i}-\overset{―}{Y}\right)}^{2}}} \end{array}$$ (18)

Where, \(ρ\)represents the Spearman rank correlation coefficient, which indicates the correlation between variables \(X\) and \(Y\), with a value range of [−1, 1]. \(ρ>0\), represents positive correlation, \(ρ<0\), represents negative correlation, \(ρ=0\), represents no correlation. $\backslash (X_{i}\backslash )$ and $\backslash (Y_{i}\backslash )$ represent the values of variables \(X\) and \(Y\) for the i-th data point, where i = 1,2,…,n. $\backslash (\overset{―}{X}\backslash )$and $\backslash (\overset{―}{Y}\backslash )$ are the sample means of variables \(X\) and \(Y\), respectively.

#### Geographically Weighted Regression (GWR).

The ESs trade-offs/synergies were quantified spatially using GWR, and the formula is as follows [42]:


$$\begin{array}{c} Y_{i}=\beta_{0}(U_{i},V_{i})+\sum_{i=1}^{k}\beta_{k}(U_{i},V_{i})X_{ik}+\varepsilon_{i} \end{array}$$ (19)

Where, $\backslash ((U_{i},\;V_{i})\backslash )$ denotes the geographic coordinates of the i^th^ raster; $\backslash (Y_{i}\backslash )$ denotes the measured value of i^th^ dependent variable Y at the spatial location; $\backslash (X_{ik}\backslash )$ denotes the measured value of the independent variable dataset $\backslash (X_{ik}\backslash )$ at the spatial location; k denotes the total number of independent variables; $\backslash (\beta_{0}(U_{i},\;V_{i})\backslash )$ denotes the constant at $\backslash ((U_{i},\;V_{i})\backslash )$; $\backslash (\beta_{k}(U_{i},V_{i})\backslash )$ denotes the value of continuous function at the i^th^ grid point; and $\backslash (\varepsilon_{i}\backslash )$ denotes the random error.

## Results

### Multi-scenario simulation of LC

#### MOP-based forecasts of LC demand.

**Fig 3:**
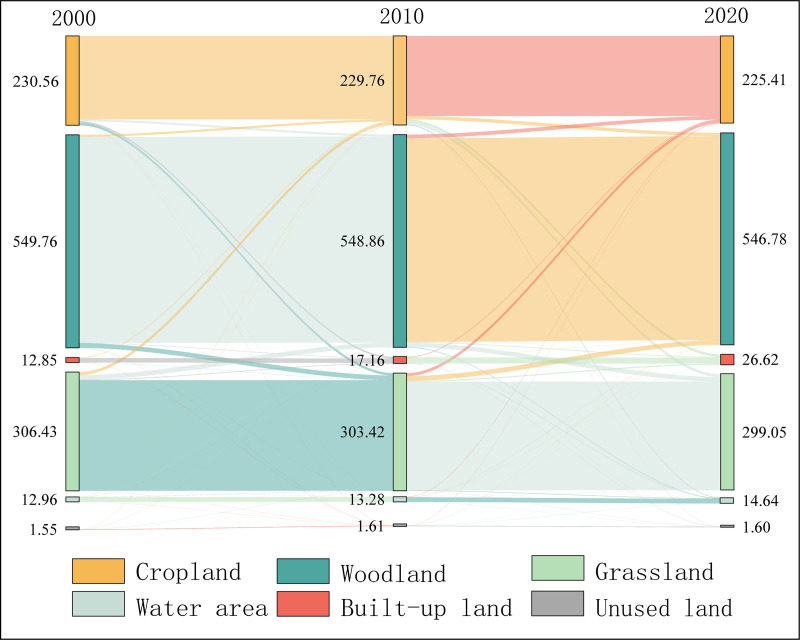
Land use transfer matrix for CYUA, 2000-2020 (hm^2^).

The analysis of LC data in CYUA between 2000 and 2020 indicated that the general trend of LUC in CYUA during the 20 years was a reduction in the area of cropland, woodland and grasslands, and a rise in the area of waters and built-up land, and a stable area of unused land (Supplementary Material, Appendix1 S1 Fig and Fig 3). According to the calculations following the aforementioned equations (1)–(13), the area of each type of land use under various scenarios in 2030 was obtained and compared with those in 2020, and it was found that: cropland decreases under the NIS and EPS scenarios, and increased in the EDS scenario; woodland land decreased under the NIS and EDS scenarios, and increased under the EPS scenario; grassland decreased to varying degrees under all three scenarios, with the most obvious decrease up to 69799.8 ha under the EDS scenario; waters increases in all three scenarios; and built-up land showed a similar trend to that of waters, with highest increase of 100826.6 ha under the EDS scenario, followed by an increase of 83346.93 ha under the NIS scenario. All the data is detailed in Table 3.

**Table 3:** Area of different types of land use (hm^2^) in CYUA in 2030 under different scenarios.

Land type	In 2020	In 2030		
		NIS	EPS	EDS
**Cropland**	2254108.23	2220871.14	2126061	2277959
**Woodland**	5467753.62	5445654.12	5522431	5413076
**Grassland**	2990462.76	2950164.63	2979666	2920663
**Water area**	146419.92	158747.49	164919	146419.9
**Built-up land**	266245.65	349592.58	332113	367072.2
**Unused land**	16049.16	16009.38	15849.3	15849.3

#### Multi-scenario simulation of LC based on MOP-PLUS model.

With various data from multiple sources, including data on LC, topography and socio-economy in CYUC in 2000, 2010 and 2020, the simulation of land use in CYUA in 2020 was performed by the PLUS model, and then a comparison between the simulation data (Supplementary Material, Appendix1 S2b Fig.)and the actual data in 2020(Supplementary Material, Appendix1 S2a Fig.) was carried out. The simulation data showed an overall accuracy of 91.97% and a kappa coefficient of 0.876, indicating that the model meets the accuracy requirements of this study.

**Fig 4:**
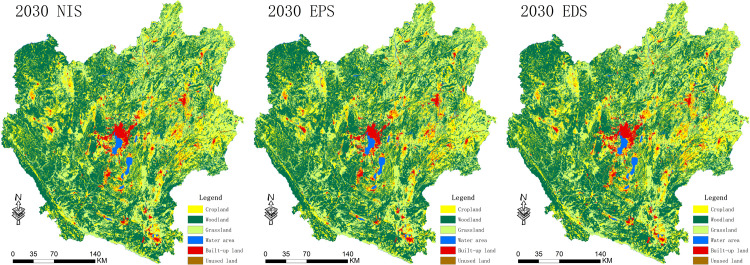
Spatial distribution of different land use scenarios for CYUA 2030.

Based on the land use maps from 2000 to 2020, we predicted the land use spatial distribution for 2030 under the NIS, EPS, and EDS scenarios (Fig 4). It is found that the dominated type of land use in CYUA under all the three scenarios is woodland, which is largely distributed in the highland mountainous regions in the western and southern areas, and is an important ecological barrier in Yunnan Province, with a proportion of approximately 49.08% of the total area; followed by grassland with an area proportion of approximately 26.84%, which is largely distributed in Kunming City, Qujing City, and Honghe Prefecture; cropland is largely distributed in blocks in the plains of CYUA, with an area proportion of approximately 20.23%; waters are mainly dominated by the six highland lakes distributed in the center of CYUA; built-up land is mainly centered in Kunming City, and the rest is distributed in the form of blocks in the urban centres and their surroundings; and the unused land accounts for a very small proportion of 0.2%, which is mainly dominated by the high altitude snow-covered mountains. Furthermore, the simulations of LC under the various scenarios indicated an increase in built-up land and waters in 2030 in comparison to that in 2020; specifically, the expansion of built-up land is mainly due to encroaching on cropland and forests, and the expansion of waters is due to spreading centered around the lakes.

### Changes in ESs and trade-off/ synergies for multi-scenario simulations

#### Analysis of changes in CYUA ESs for multi-scenario simulations.

**Fig 5:**
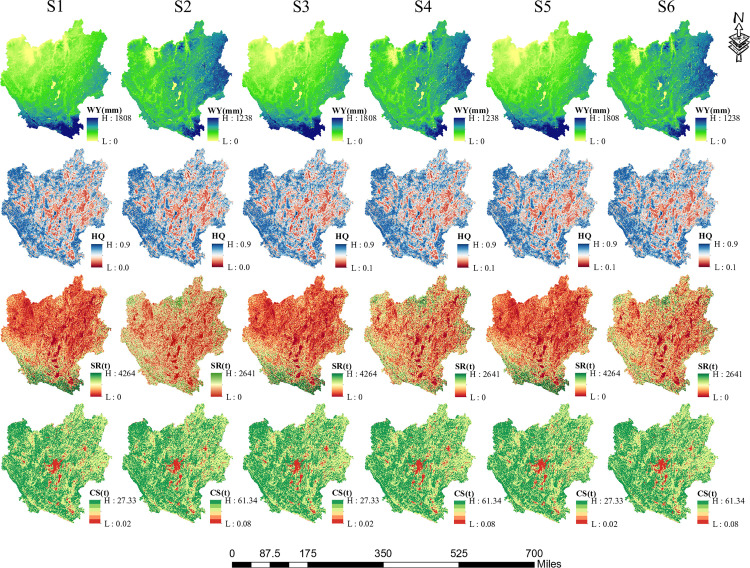
Spatial distribution of ESs in CYUA ESs in 2030 under various scenarios.

The MOP-PLUS-RCP and InVEST models were coupled to predict the future (2030) changes of WY, HQ, SR, and CS services in the CYUA under six different scenarios, and the results are provided in Table 4 and Fig 5 and described as follows:

**Table 4:** Number of simulated ESs in CYUA in 2030under various scenarios.

	S1	S2	S3	S4	S5	S6
**WY(m**^**3**^**·hm**^**-2**^)	3445.17	5300.907	3435.627	5288.22	3453.037	5312.13
**HQ**	0.606	0.606	0.610	0.610	0.604	0.604
**SR (t·hm**^**-2**^)	2135.3354	3023.144	2135.5183	3023.378	2135.122	3022.788
**CS (t·hm**^**-2**^)	244.05049	552.3114	244.96495	554.4611	243.6671	551.3895

(1) WY and characteristics of its spatial distribution: under different scenarios, Scenario 6 was projected to have the highest WY (5312.13 m^3^/ha), followed by Scenario 2 (5300.90 m^3^/ha) and Scenario 4 (5288.22 m^3^/ha). For climate scenarios, the WY of RCP8.5 is higher than that of RCP4.5, which may be related to the high precipitation and low temperature of RCP8.5. For land use scenarios, the WY of EDS scenario was larger than that of the EPS and NIS2 scenarios, and the main explanation to this is the increase in cropland under the EDS scenario, which mitigates the impacts of reduced woodlands and grasslands. With respect to spatial distribution, it was roughly the same under the same climate scenario. For RCP4.5 (in Fig 5 S1, S3 and S5), the WY showed a trapezoidal distribution, with gradual increase from north to south; for RCP8.5 (in Fig 5 S2, S4 and S6), the distribution of WY was low in the northwestern region, and high in the eastern and southern regions. It should be noted that the lower reaches of the Yuan River in the south were always the main water-producing area in all scenarios.
(2) HQ and characteristics of its spatial distribution: From the average HQ of various scenarios, the effect of land use on HQ was greater than that of climate. Therefore, HQ showed no significant change under different climate scenarios. While for HQ under LC scenarios, it was ranked from high to low as follows: EPS (0.610)> NIS (0.606)> EDS (0.604), all of which are of high HQ, and this is closely correlated with the high forest coverage rate of the region. Therefore, due to more attention paid to ecological protection, which resulted in increased woodland, the EPS scenario showed the best HQ. With respect to spatial distribution, all six scenarios showed a same pattern of high in the western region and low in the centre. Changes in HQ were largely concentrated in the center, where is densely populated and highly urbanised, indicating that HQ was directly affected by the increase in built-up land.
(3) SR and characteristics of its spatial distribution: among the various scenarios, S4 showed the best SR, followed by S2 and S6. Compared with that in 2020, the SR of all the six scenarios has increased, with the EPS scenario showing the most significant increase. With respect to spatial distribution, the high SR was mainly located in the lower reaches of the Yuan River in the south, while the rest of the study area showed different low values, especially in the central area, with a downtrend from west to east. This indicates that the high vegetation cover in the south plays a positive role in soil conservation, while in the west, due to the high altitude difference and dense steep slopes, the soil and water conservation capacity is compromised even if there is a high vegetation cover. Although the terrain in the central region is relatively flat, most of the region is an urban area under development, so the soil conservation capacity is low, indicating that continued urban expansion has led to encroachment on ecological land.
(4) CS and characteristics of its spatial distribution: the order of CS under different scenarios is as follows: S4 > S2 > S6 > S3 > S1 > S5. It can be seen that the CS capacity under the RCP8.5 is significantly greater than that under the RCP4.5, and that the EPS scenario has the most prominent CS capacity among the various LC scenarios. From the perspective of their spatial distributions, the CS capacity and HQ of S6 showed a highly consistent overall pattern of high in the west and low in the centre. In addition, the CS reduction mainly occurs in the periphery of the central cities. The above findings suggest that urban development in the future will occupy a large number of lands with high CS, such as agricultural land and forests, resulting in decreased CS in the region.

#### Changes in ES trade-offs/synergies under different scenarios.

Using the Spearman correlation, the trade-offs/synergies between ESs under various scenarios was analyzed to explore the changes in relationship between ESs under various scenarios in 2020 and 2030. As shown in Table 5, the results indicated a positive correlation between WY-SR, HQ-SR, HQ-CS and SR-CS, reflecting a synergistic relationship, and a negative correlation between WY-HQ and WY-CS, indicating a trade-off relationship, in both 2020 and 2030 across different scenarios. Compared to 2020, under the RCP4.5 climate model (S1, S3 and S5), the trade-off between WY-HQ and WY-CS in 2030 was weakened, while the synergistic relationship between WY-SR was strengthened, particularly in S5, where the increase was approximately 0.22. The synergistic effects between CS-SR and CS-HQ are weakening and increasing, respectively; under the RCP8.5 climate model (S2, S4 and S6), the synergistic relationship between CS-SR and CS-HQ were weakened and increased, respectively, which are in exactly the opposite way to that of the RCP4.5 model. It is noteworthy that the HQ-SR synergies showed different degrees of weakening in all six future scenarios. Under the same climate scenarios, the trade-offs between WY-HQ and WY-CS and the synergies between WY-SR, HQ-CS, and SR-CS were higher in the EDS scenario than in the EPS and NIS scenarios, and the HQ-SR synergy was highest in the EPS scenario.

**Table 5:** ES trade-offs/synergies under various scenarios.

Trade-offs/ synergies	In 2020	In 2030					
		S1	S2	S3	S4	S5	S6
**WY-HQ**	−0.199	−0.101	−0.281	−0.095	−0.278	−0.106	−0.286
**WY-SR**	0.111	0.334	0.016	0.335	0.015	0.336	0.017
**WY-CS**	−0.205	−0.104	−0.257	−0.099	0.255	−0.112	−0.265
**HQ-SR**	0.338	0.333	0.336	0.334	0.336	0.331	0.336
**HQ-CS**	0.782	0.784	0.784	0.779	0.779	0.789	0.789
**SR-CS**	0.286	0.282	0.294	0.284	0.294	0.285	0.297

**Fig 6:**
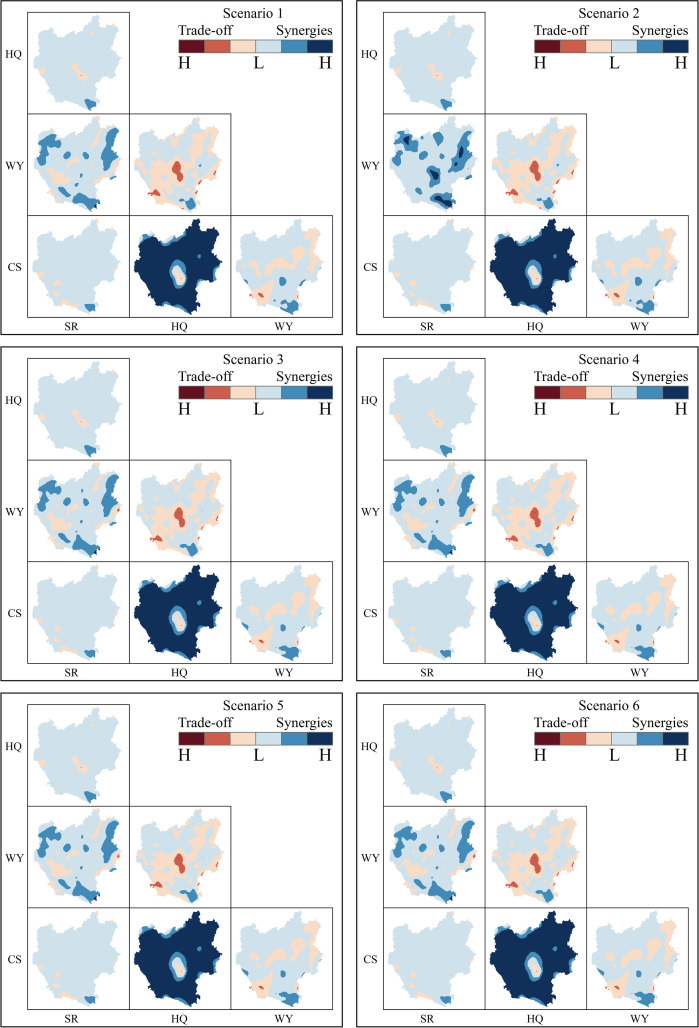
Spatial distribution of ES trade-offs/synergies under different scenarios.

For the purpose of further elucidating the significant spatial heterogeneity of ESs trade-offs/synergies due to geographic differences in highland mountainous regions, the spatial layout of such trade-offs/synergies was obtained at the raster scale using GWR analysis. As presented in Fig 6, the results indicated that the trade-offs/synergies between four pairs of ESs were consistent in spatial distribution under all the six scenarios; SR-HQ spatial effects showed a weak synergy in general, but showed a weak trade-off in the highly urbanised region in the central area; SR-WY showed a synergy in areas distributed in blocks in Qujing City, Chuxiong City, and Honghe Prefecture, and showed a trade-off relationship in areas mainly distributed in Yuxi City; SR-CS showed a weak synergistic relationship in the whole area excepting the southwestern region, in where it showed a weak trade-off relationship; HQ-WY showed a strong synergy in areas largely located in the north and the south, and a weak trade-off relationship in areas mainly distributed in the west and the northeast and particularly evident in the central region; HQ-CS showed a weak synergy in the western and northeastern regions, and a strong synergistic relationship in the rest; WY-CS showed a weak synergy in the northern and northeastern regions and a relatively strong synergy in the south of Honghe Prefecture, and a weak trade-off in the southwest of Yuxi City, Kunming City, and the east of Qujing City.

## Discussion

The purpose of this study was to systematically understand the influencing mechanisms of climate and land use changes on ecosystem services (ESs). This includes analyzing their future trajectories and the trade-offs/synergies between them. To achieve this, a coupled MOP-PLUS-RCP model was established. Using this model, land use change (LUC) in CYUA for 2030 was simulated under three different scenarios: NIS, EPS, and EDS. Next, the simulations were combined with two future climate models, RCP4.5 and RCP8.5. This led to the creation of six LULC-RCP scenarios. Under these scenarios, the future changes in four key ESs (WY, CS, SR, HQ) were quantitatively assessed using the InVEST model. Additionally, spatio-temporal trade-offs and synergies in ESs were explored through Spearman correlation analyses and Geographically Weighted Regression (GWR) at the grid scale.

### Multi-scenario simulation of LC

The assignment of LC planning is a stepwise process from top to bottom, which is manifested by focusing on the scale and proportion of LC and neglecting the overall spatial layout and regional integration, thereby resulting in generally the problem of spatial conflict between the structure-quantity synergy of top-down LC and the bottom-up layout of LC, when simulating the future LC [43]. In this study, the MOP-PLUS coupled model was constructed for simulating the quantity-structure and spatial distribution of LC of multiple scenarios in the future, and according to the principle of maximising economic and ecological benefits, EDS and EPS scenarios were set up; moreover, NIS scenario was set up as a reference scenario based on the historical trend of development. The MOP algorithm can predict the future quantity demand of LC from top to bottom according to the governmental policy requirement and the actual situation of CYUA. In addition, the PLUS model combines the RF and the bottom-up CA allocation mechanism to reasonably allocate the quantities of LC under each scenario, and to simulate the types of LC under various scenarios. Therefore, the combination of the MOP algorithm and the PLUS model can solve the synergistic problem in simulating quantities and spatial patterns of LC under various scenarios, which lays the foundation for subsequent researches.

### Influences of changes in LC and climate on ESs and trade-offs/synergies between them

**Fig 7:**
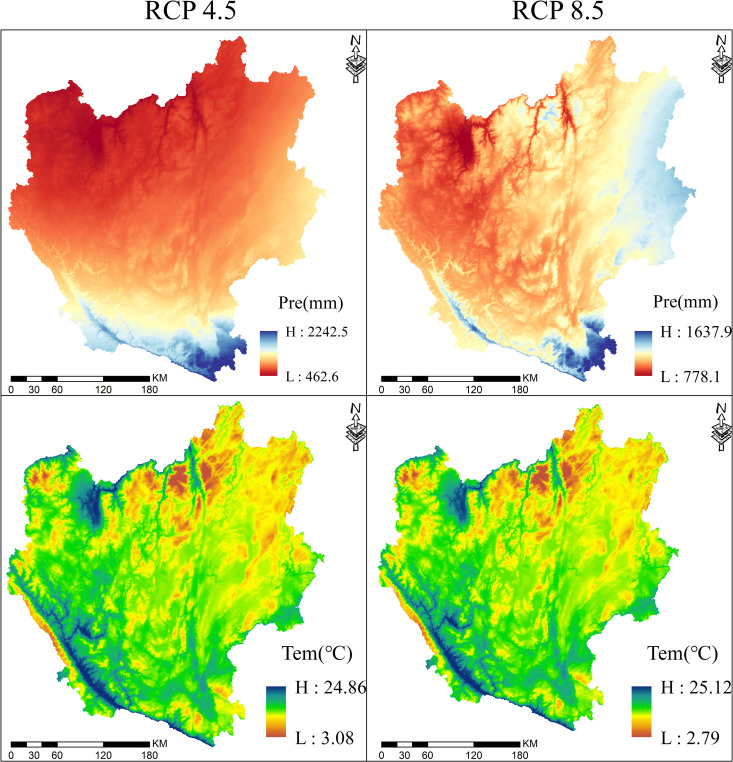
Spatial distribution of precipitation and temperature under different climate models.

The LUC and climate have been recognised as critical influencing factors on changes in ESs, and such changes will increasingly affect the dynamics of ESs in the future [44]. Based on the spatio-temporal distributions of ESs under two climate models (Fig 7) and six scenarios(Fig 5), it was found that the average values of WY, SR, and CS in the RCP8.5 climate model were higher than those in the RCP4.5 climate model; whereas, the precipitation and air temperature gradually increased spatially from northwest to southeast, as well as the WY and SR. The explanation for this is that regions with higher precipitation and air temperature typically have denser vegetation cover, which more effectively reduces the risk of soil erosion and directly influences the recharge of surface runoff and groundwater flow [45]. Moreover, the effect of precipitation on WY may be partially offset by a large water consumption by high vegetation cover due to transpiration and by high actual evapotranspiration attributed to water retention and evapotranspiration through the forest canopy [46]. Thus HQ-SR, WY-SR showed synergistic effects. According to previous studies, there is a positive correlation of carbon density with precipitation and a weak correlation of carbon density with air temperature [37]. Since in the two climate models of RCP4.5 and RCP8.5, the average annual precipitation was 851.55 mm and 1080.60 mm, respectively, and the average annual temperature was 16.55°C and 16.47°C, respectively, the carbon density of various types of land use in the RCP8.5 is remarkably higher than that in the RCP4.5, This result is consistent with the findings of Zhang et al. in the Xijiang River Basin [31]. The higher the vegetation cover, the higher the CS, and in addition, a higher vegetation generally means a richer biodiversity and higher soil nutrients, and Similar to the findings of Wang et al [47]. Therefore, HQ-CS and SR-CS showed a relatively high synergistic relationship. However, precipitation promotes both the growth of vegetation and continuous loss of nutrients from the soil, especially in the northwestern part of the region, which has large elevation differences and steep slopes, leading to serious soil nutrient runoff despite high vegetation cover, while the eastern part belongs to the typical karstic ground, with sparse vegetation and serious rainfall erosion [48]. Therefore, WY-HQ and WY-CS showed a weak negative correlation in these regions.

In term of LC, under the same climatic conditions, the main changes of the four ESs in the NIS, EPS, and EDS scenarios were concentrated in Kunming City and its periphery, such as Yuxi City, Qujing City, and Chuxiong City, etc., which are regions where the increasing population and accelerated urbanisation have resulted in the sustained expansion of built-up land into the surrounding croplands, woodland, grasslands, and waters, especially in the EDS scenario, which may bring a number of ecological issues, such as the reduction of biodiversity and carbon sink capacity due to the reduction of forests and grasslands; the weakened ability of urban ecosystems to purify air and regulate the hydrological cycle due to reduction of waters [49]. The study also found that areas with expanding built-up land tend to be areas with more severe trade-offs between ESs. These findings can help decision makers to implement ecological projects in the future.

This study can strengthen stakeholders’ understanding on the mutual effects between ecosystem performance indicators under various scenarios. For future policies on land use regulation, it is necessary to take proactive interventions to steer land use towards sustainability. The main recommendations are as follows: ① in term of climate, strengthening climate monitoring and developing appropriate management policies for different patterns of global climate change with use of prediction models; ② in terms of ecological protection, enforcing the closure of high energy-consuming and high-polluting factories, prohibiting grazing, and preventing deforestation activities of land reclamation and mining, etc. In addition, it is recommended to implement actively restorations to general ecoregions such as reforestation and afforestation, thereby strengthen ecological protection.

### Shortcomings and prospects

In terms of land prediction, although the MOP method can integrate the policy planning and the actual situation of CYUA into the simulation of future land use, it is unable to constrain the natural geographic conditions during the LUC, such as precipitation, temperature and other climatic conditions that may change the conversion of land use; in the computation and selection of biophysical parameters for various modules of the InVEST model, some of the required data and parameters are sourced from existing literature and may differ from the actual situation of CYUA, leading to difference between the results and the actual situation or existing research results. Although different scenarios were set up herin to explore the influences of land use and climate in the future on ESs, the probability of future development under various scenarios of climate and land use could not be fully explained, and it is necessary to investigate the dynamic changes of trade-offs and synergies between ESs and the relevant driving mechanisms under various climatic scenarios and at different scales in the future research, so as to strengthen the comprehensive evaluation on the trade-offs and synergies between ESs in CYUA, and to offer proposals on ES zoning and regulation of anthropogenic activities.

## Conclusion

(1) In simulations under all the scenarios, the area of both waters and built-up land increased in the CYUA. For waters, the highest increase rate of area was in the EPS scenario, followed by the NIS and SD scenarios; for built-up land, the highest increase rate was in the EDS scenario, followed by the NIS scenario.
(2) The spatial heterogeneity of ESs was strong in CYUA. Specifically, the spatial distribution of WY and SR was in general low in the northwestern region and high in the southeastern region; and for HQ and CS, it was high in general in the western and surrounding regions and low in the eastern and central regions. In all the land use scenarios, the ESs of RCP8.5 climate model were higher than those of RCP4.5 climate model. While under the two different climate models, the order of WY was EDS > NIS > EPS, and the order of HQ, SR and CS was EPS > NIS > EDS.
(3) The changes in ES trade-offs/synergies varied from different scenarios. Among them, WY-SR, HQ-SR, HQ-CS, SR-CS presented a positive correlation, and WY-HQ and WY-CS presented a negative correlation. Compared with 2020, WY-HQ, WY-CS, HQ-SR, and SR-CS in 2030 under Scenarios 1, 3, and 5 showed a weakening trend, and WY-SR showed a strengthening trend; while the behaviors under Scenarios 2, 4, and 6 were exactly the opposite.
(4) Under all the six different scenarios, the spatial distribution of ES trade-offs/synergies showed no difference for the four ESs. The spatial effects of SR-HQ, SR-CS, and WY-CS were generally weak synergistic relationships; the spatial effects of HQ-CS were generally weak trade-offs; and the spatial effects of HQ-WY and CS-WY showed weak synergies in the northern and southern region of CYUA, and weak synergies in the central region.

## Supporting information

S1 Fig Land use data from 2000 to 2020. (TIF)

S2 Fig Simulated land use data for 2020. (a) versus actual land use data (b).
(TIF)

## References

[1] Daily GC. In: Robin L, S枚rlin S, Warde P ed. Nature is services: societal dependence on natural ecosystems (1997). Yale University Press; 2013. p. 454–64.

[2] Arowolo AO, Deng X, Olatunji OA, Obayelu AE. Assessing changes in the value of ecosystem services in response to land-use/land-cover dynamics in Nigeria. Sci Total Environ. 2018;636:597–609. doi: 10.1016/j.scitotenv.2018.04.277 29723833

[3] Yang Y, Wang K, Liu D, Zhao X, Fan J. Effects of land-use conversions on the ecosystem services in the agro-pastoral ecotone of northern China. J Cleaner Prod. 2020;249:119360. doi: 10.1016/j.jclepro.2019.119360

[4] Wenbo X, Hengzhou X, Xiaoyan L, Hua Q, Ziyao W. Ecosystem services response to future land use/cover change (LUCC) under multiple scenarios: a case study of the Beijing-Tianjin-Hebei (BTH) region, China. Technol Forecast Soc Change. 2024;205:123525. doi: 10.1016/j.techfore.2024.123525

[5] Pei H, Liu M, Shen Y, Xu K, Zhang H, Li Y, et al. Quantifying impacts of climate dynamics and land-use changes on water yield service in the agro-pastoral ecotone of northern China. Sci Total Environ. 2022;809:151153. doi: 10.1016/j.scitotenv.2021.151153 34688740

[6] Hoyer R, Chang H. Assessment of freshwater ecosystem services in the Tualatin and Yamhill basins under climate change and urbanization. Applied Geography. 2014;53:402–16. doi: 10.1016/j.apgeog.2014.06.023

[7] Grimm NB, Chapin FS III, Bierwagen B, Gonzalez P, Groffman PM, Luo Y, et al. The impacts of climate change on ecosystem structure and function. Frontiers in Ecol Environ. 2013;11(9):474–82. doi: 10.1890/120282

[8] Weiskopf SR, Rubenstein MA, Crozier LG, Gaichas S, Griffis R, Halofsky JE, et al. Climate change effects on biodiversity, ecosystems, ecosystem services, and natural resource management in the United States. Sci Total Environ. 2020;733:137782. doi: 10.1016/j.scitotenv.2020.137782 32209235

[9] Wang Y, Zhao J, Fu J, Wei W. Effects of the grain for green program on the water ecosystem services in an arid area of China—using the Shiyang River Basin as an example. Ecol Indic. 2019;104:659–68. doi: 10.1016/j.ecolind.2019.05.045

[10] Valencia TA, Tiwari C, Atkinson SF. Progress in ecosystem services research: a guide for scholars and practitioners. Ecosyst Serv. 2021;49:101267. doi: 10.1016/j.ecoser.2021.101267

[11] Liu M, Xiong Y, Zhang A. Multi-scale telecoupling effects of land use change on ecosystem services in urban agglomerations --A case study in the middle reaches of Yangtze River urban agglomerations. J Cleaner Prod. 2023;415:137878. doi: 10.1016/j.jclepro.2023.137878

[12] Yang Y, Yuan X, An J, Su Q, Chen B. Drivers of ecosystem services and their trade-offs and synergies in different land use policy zones of Shaanxi Province, China. J Cleaner Prod. 2024;452:142077. doi: 10.1016/j.jclepro.2024.142077

[13] Wu Y, Zhang X, Li C, Xu Y, Hao F, Yin G. Ecosystem service trade-offs and synergies under influence of climate and land cover change in an afforested semiarid basin, China. Ecol Eng. 2021;159:106083. doi: 10.1016/j.ecoleng.2020.106083

[14] Chen W, Chi G, Li J. The spatial association of ecosystem services with land use and land cover change at the county level in China, 1995-2015. Sci Total Environ. 2019;669:459–70. doi: 10.1016/j.scitotenv.2019.03.139 30884268

[15] Xiao J, Song F, Su F, Shi Z, Song S. Quantifying the independent contributions of climate and land use change to ecosystem services. Ecol Indic. 2023;153:110411. doi: 10.1016/j.ecolind.2023.110411

[16] Guo M, Ma S, Wang L-J, Lin C. Impacts of future climate change and different management scenarios on water-related ecosystem services: a case study in the Jianghuai ecological economic Zone, China. Ecological Indicators. 2021;127:107732. doi: 10.1016/j.ecolind.2021.107732

[17] Sun L, Yu H, Sun M, Wang Y. Coupled impacts of climate and land use changes on regional ecosystem services. J Environ Manage. 2023;326(Pt A):116753. doi: 10.1016/j.jenvman.2022.116753 36399886

[18] Assaf G, Assaad RH. Modeling the impact of land use/land cover (LULC) factors on diurnal and nocturnal Urban Heat Island (UHI) intensities using spatial regression models. Urban Climate. 2024;55:101971. doi: 10.1016/j.uclim.2024.101971

[19] Saha P, Mitra R, Chakraborty K, Roy M. Application of multi layer perceptron neural network Markov chain model for LULC change detection in the Sub-Himalayan North Bengal. Remote Sens Appl: Soc Environ. 2022;26:100730. doi: 10.1016/j.rsase.2022.100730

[20] Yang K, Hou H, Li Y, Chen Y, Wang L, Wang P, et al. Future urban waterlogging simulation based on LULC forecast model: a case study in Haining City, China. Sustainable Cities Soc. 2022;87:104167. doi: 10.1016/j.scs.2022.104167

[21] Zhang Y, Liu Y, Zhang Y, Liu Y, Zhang G, Chen Y. On the spatial relationship between ecosystem services and urbanization: a case study in Wuhan, China. Sci Total Environ. 2018;637–638:780–90. doi: 10.1016/j.scitotenv.2018.04.396 29758433

[22] Sun Y, Liu S, Dong Y, An Y, Shi F, Dong S, et al. Spatio-temporal evolution scenarios and the coupling analysis of ecosystem services with land use change in China. Sci Total Environ. 2019;681:211–25. doi: 10.1016/j.scitotenv.2019.05.136 31103659

[23] Ai M, Chen X, Yu Q. Spatial correlation analysis between human disturbance intensity (HDI) and ecosystem services value (ESV) in the Chengdu-Chongqing urban agglomeration. Ecol Indic. 2024;158:111555. doi: 10.1016/j.ecolind.2024.111555

[24] Zhang Y, Zheng M, Qin B. Optimization of spatial layout based on ESV-FLUS model from the perspective of “Production-Living-Ecological”: a case study of Wuhan City. Ecol Model. 2023;481:110356. doi: 10.1016/j.ecolmodel.2023.110356

[25] Mukhopadhyay A, Hati JP, Acharyya R, Pal I, Tuladhar N, Habel M. Global trends in using the InVEST model suite and related research: a systematic review. Ecohydrol Hydrobiol. 2025;25(2):389–405. doi: 10.1016/j.ecohyd.2024.06.002

[26] Liu Y, Jing Y, Han S. Multi-scenario simulation of land use/land cover change and water yield evaluation coupled with the GMOP-PLUS-InVEST model: a case study of the Nansi Lake Basin in China. Ecol Indic. 2023;155:110926. doi: 10.1016/j.ecolind.2023.110926

[27] Rachid L, Elmostafa A, Mehdi M, Hassan R. Assessing carbon storage and sequestration benefits of urban greening in Nador City, Morocco, utilizing GIS and the InVEST model. Sustainable Futures. 2024;7:100171. doi: 10.1016/j.sftr.2024.100171

[28] Gang L, Gaoming J, Yonggeng L, Meizhen L. Biomass carbon storage and net primary production in different habitats of Hunshandake Sandland, China. Acta Ecologica Sinica. 2011;31(4):217–24. doi: 10.1016/j.chnaes.2011.04.006

[29] Popp A, Calvin K, Fujimori S, Havlik P, Humpenöder F, Stehfest E, et al. Land-use futures in the shared socio-economic pathways. Global Environ Change. 2017;42:331–45. doi: 10.1016/j.gloenvcha.2016.10.002

[30] Wang Z, Li X, Mao Y, Li L, Wang X, Lin Q. Dynamic simulation of land use change and assessment of carbon storage based on climate change scenarios at the city level: a case study of Bortala, China. Ecol Indic. 2022;134:108499. doi: 10.1016/j.ecolind.2021.108499

[31] Zhang Y, Wu T, Song C, Hein L, Shi F, Han M, et al. Influences of climate change and land use change on the interactions of ecosystem services in China’s Xijiang River Basin. Ecosyst Serv. 2022;58:101489. doi: 10.1016/j.ecoser.2022.101489

[32] Li J, Guldmann J-M, Gong J, Su H. Urban growth boundaries optimization under low-carbon development: Combining multi-objective programming and patch cellular automata models. J Environ Manage. 2023;340:117934. doi: 10.1016/j.jenvman.2023.117934 37105107

[33] Xie GD, Zhang CX, Zhang LM, Chen WH, Li SM. Improvement of the evaluation method for ecosystem service value based on per unit area. J Nat Res. 2015;30(08):1243–54.

[34] Liang X, Guan Q, Clarke KC, Liu S, Wang B, Yao Y. Understanding the drivers of sustainable land expansion using a patch-generating land use simulation (PLUS) model: a case study in Wuhan, China. Comp Environ Urban Syst. 2021;85:101569. doi: 10.1016/j.compenvurbsys.2020.101569

[35] Xie X, Sun B, Zhou HZ, Li ZP, Li A. Organic carbon density and storage in soils of China and spatial analysis. Acta Pedologica Sinica. 2004;41:35–43.

[36] Qiu H, Hu B, Zhang Z. Impacts of land use change on ecosystem service value based on SDGs report--Taking Guangxi as an example. Ecol Indic. 2021;133:108366. doi: 10.1016/j.ecolind.2021.108366

[37] Xiang M, Wang C, Tan Y, Yang J, Duan L, Fang Y, et al. Spatio-temporal evolution and driving factors of carbon storage in the Western Sichuan Plateau. Sci Rep. 2022;12(1):8114. doi: 10.1038/s41598-022-12175-8 35581278 PMC9114110

[38] He C, Zhang D, Huang Q, Zhao Y. Assessing the potential impacts of urban expansion on regional carbon storage by linking the LUSD-urban and InVEST models. Environ Model Softw. 2016;75:44–58. doi: 10.1016/j.envsoft.2015.09.015

[39] Dash SS, Maity R. Effect of climate change on soil erosion indicates a dominance of rainfall over LULC changes. J Hydrol: Reg Stud. 2023;47:101373. doi: 10.1016/j.ejrh.2023.101373

[40] Huang Y, Wu J. Spatial and temporal driving mechanisms of ecosystem service trade-off/synergy in national key urban agglomerations: a case study of the Yangtze River Delta urban agglomeration in China. Ecol Indic. 2023;154:110800. doi: 10.1016/j.ecolind.2023.110800

[41] Li Q, Li W, Wang S, Wang J. Assessing heterogeneity of trade-offs/synergies and values among ecosystem services in Beijing-Tianjin-Hebei urban agglomeration. Ecol Indic. 2022;140:109026. doi: 10.1016/j.ecolind.2022.109026

[42] Liu Q, Qiao J, Li M, Huang M. Spatiotemporal heterogeneity of ecosystem service interactions and their drivers at different spatial scales in the Yellow River Basin. Sci Total Environ. 2024;908:168486. doi: 10.1016/j.scitotenv.2023.168486 37952663

[43] Li C, Wu Y, Gao B, Zheng K, Wu Y, Li C. Multi-scenario simulation of ecosystem service value for optimization of land use in the Sichuan-Yunnan ecological barrier, China. Ecol Indicat. 2021;132:108328. doi: 10.1016/j.ecolind.2021.108328

[44] Zhang H, Jiang C, Wang Y, Wang J, Li C, Yang Z, et al. Improving the integrated efficacy of ecosystem restoration efforts by linking land degradation neutrality to ecosystem service enhancement from a spatial association perspective. Ecol Eng. 2022;181:106693. doi: 10.1016/j.ecoleng.2022.106693

[45] Lin Q, Wu Z, Singh VP, Sadeghi SHR, He H, Lu G. Correlation between hydrological drought, climatic factors, reservoir operation, and vegetation cover in the Xijiang basin, South China. J Hydrol. 2017;549:512–24. doi: 10.1016/j.jhydrol.2017.04.020

[46] Liu S, Wang J, Wei J, Wang H. Hydrological simulation evaluation with WRF-Hydro in a large and highly complicated watershed: the Xijiang river basin. J Hydrol: Reg Stud. 2021;38:100943. doi: 10.1016/j.ejrh.2021.100943

[47] Wang Z, Liu S, Su Y. Spatiotemporal evolution of habitat quality and its response to landscape patterns in karst mountainous cities: a case study of Guiyang City in China. Environ Sci Pollut Res Int. 2023;30(53):114391–405. doi: 10.1007/s11356-023-30420-z 37861839

[48] Feng T, Chen H, Polyakov VO, Wang K, Zhang X, Zhang W. Soil erosion rates in two karst peak-cluster depression basins of northwest Guangxi, China: comparison of the RUSLE model with 137Cs measurements. Geomorphology. 2016;253:217–24. doi: 10.1016/j.geomorph.2015.10.013

[49] Zhang Z, Li J, Lu Y, Yang L, Hu Z, Li C, et al. Temporal and spatial changes in land use and ecosystem service value based on SDGs’ reports: a case study of Dianchi Lake Basin, China. Environ Sci Pollut Res Int. 2023;30(11):31421–35. doi: 10.1007/s11356-022-24263-3 36449234

