# Supplementary figures and images for: Land use and climate change-based multi-scenario simulation of ecosystem service trade-offs/synergies: A case study of the central Yunnan urban agglomeration, China
Source: PLoS One. 2025 Jun 25;20(6):e0324015. doi: 10.1371/journal.pone.0324015 (PMC12193797; doi:10.1371/journal.pone.0324015)

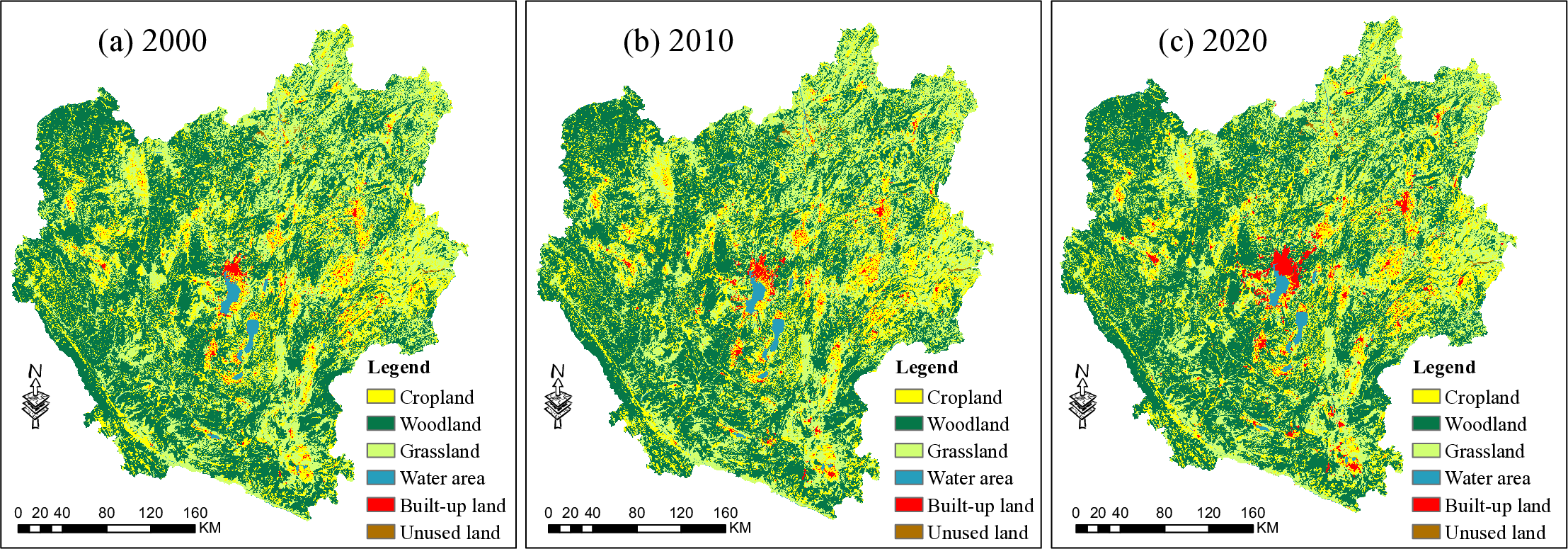

Supplement: S1 Fig — (TIF) [file pone.0324015.s001.tif]

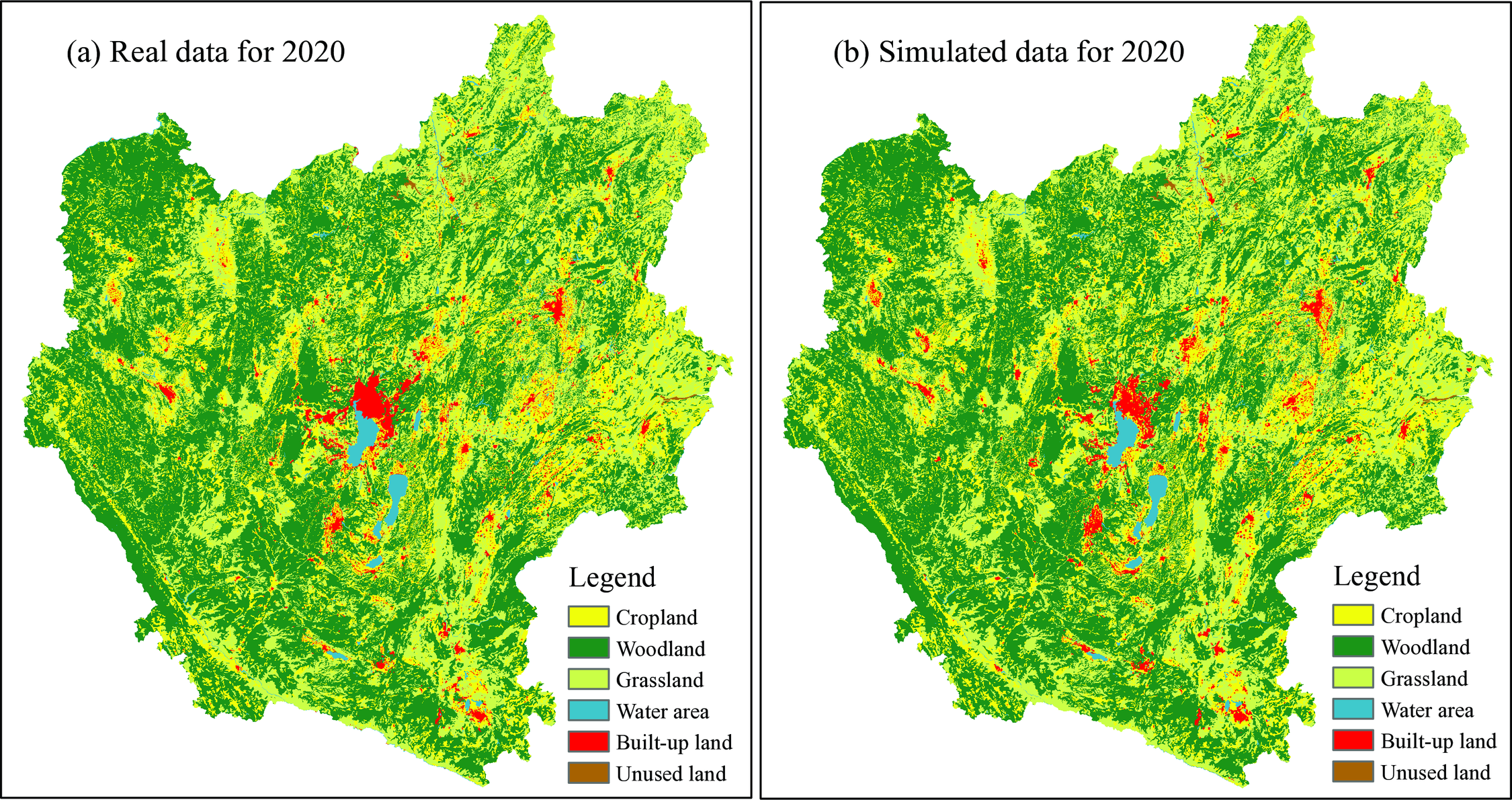

Supplement: S2 Fig — (a) versus actual land use data (b). (TIF) [file pone.0324015.s002.tif]
